# A Case Report of Under-Recognized Conditions in Pulmonary Embolism: Patent Foramen Ovale and Right Ventricular Thrombus

**DOI:** 10.7759/cureus.52535

**Published:** 2024-01-18

**Authors:** Satoshi Sera, Yuji Okazaki, Kenichiro Kashiwa, Toshihisa Ichiba

**Affiliations:** 1 Department of Emergency Medicine, Hiroshima City Hiroshima Citizens Hospital, Hiroshima, JPN

**Keywords:** right ventricular cardiac thrombus, patent foramen ovale (pfo), paradoxical embolism, renal artery infarction, acute pulmonary embolism

## Abstract

Pulmonary embolism (PE) is a potentially life-threatening condition that presents with a spectrum of clinical symptoms ranging from asymptomatic to hemodynamic instability. The early diagnosis in the emergency department is often challenging. Although the association between patent foramen ovale (PFO) and thromboembolic events in patients with PE is well-documented, the significance of the presence of PFO in patients with PE may be underrecognized. In addition, the occurrence of right ventricular thrombus (RVT) in PE is a rare but significant complication with implications for disease management. We report a case of acute-on-chronic PE with concurrent bilateral renal infarction due to a paradoxical embolus, alongside RVT. A 35-year-old male presented at our emergency department with complaints of sudden onset abdominal pain. Bilateral renal infarction was identified on a contrast-enhanced computed tomography (CT). Point-of-care ultrasound showed suggestive findings of PE and RVT. Subsequently, a pulmonary CT angiography confirmed bilateral PE, a PFO, and RVT. The patient was effectively managed with thrombolytic therapy, with extracorporeal membrane oxygenation on standby. This case highlights the need to recognize the diverse clinical manifestations of PE and the importance of considering coexisting PFO and RVT in affected patients. The diagnosis of PE can be complex when symptoms overlap with arterial thrombosis, such as renal infarction secondary to a PFO. In addition, RVT, although uncommon, is a serious complication in patients with PE that may require careful evaluation for thrombolytic or anticoagulant therapy. It is critical to consider the possibility of a PFO in all cases of PE, even in the absence of arterial embolism, and to promptly evaluate for RVT prior to initiating treatment.

## Introduction

Pulmonary embolism (PE) is a common and potentially life-threatening condition, and it has various clinical presentations, ranging from no symptoms to hemodynamic instability [1]. When PE is coexistent with other diseases, such as acute ischemic stroke, the symptoms of PE are often masked. Patent foramen ovale (PFO) is a remnant of fetal circulation and is found in approximately 25% of the general population [2]. PFO is a major cause of paradoxical embolism, including acute ischemic stroke or, in rare cases, renal infarction [3]. In patients with PE, PFO is associated with thromboembolic complications and a high mortality rate [4]. Thus, the presence of a PFO should be considered when the cause of arterial embolism is uncertain, and it may be equally important to search for a PFO in patients with PE. We present a case of sub-massive PE with a PFO, which presented with acute abdominal pain and was subsequently diagnosed as bilateral renal infarction and right ventricular thrombus (RVT).

## Case presentation

A 35-year-old man presented to our emergency department with a complaint of acute abdominal pain and vomiting. His abdominal pain was continuous, and he experienced only one episode of vomiting before admission. He did not have a medical history of constipation. He had undergone surgery on his left lower leg for an accident two years ago but had no thrombotic disorders. He had dyspnea on exertion three months before admission. On arrival, his blood pressure was 134/72 mmHg, heart rate was 120 beats per minute with sinus rhythm, respiratory rate was 28 per minute, and oxygen saturation was 85% on room air despite no dyspnea, cough, or hemoptysis. On physical examination, his abdomen was soft but tender, and there were no heart murmurs or swelling of his lower extremities. In addition, lung sounds were normal, accessory respiratory muscles were not used, and he was not cyanotic. Venous blood gas analysis revealed elevated lactic acid levels, while blood examinations indicated a marked increase in lactate dehydrogenase and D-dimer levels. The international normalized ratio of prothrombin time, antithrombin III, protein C, and protein S were all within normal ranges (Table 1).

**Table 1:** Laboratory examination ALT, alanine transaminase; AST, aspartate transferase; γ-GTP, γ-glutamyl transpeptidase; ALP, alkaline phosphatase; LDH, lactate dehydrogenase; NT-proBNP, N-terminal pro-B-type natriuretic peptide; PT-INR, international normalized ratio of prothrombin time; APTT, activated partial thromboplastin time

	Hospital admission	Reference range
White blood counts (x 10^3^/μL)	11.8	3.3-8.6
Hemoglobin (g/dL)	17.6	13.7-16.8
Platelet (x 10^4^/μL)	17.9	15.8-34.8
AST (U/L)	59	13-30
ALT (U/L)	42	10-42
γ-GTP (U/L)	58	13-64
ALP (U/L)	298	106-322
LDH (U/L)	710	124-222
Amylase (U/L)	40	44-132
Blood urea nitrogen (mg/dL)	12	8-20
Creatinine (mg/dL)	0.98	0.65-1.07
Creatinine kinase (U/L)	64	59-248
C-reactive protein (mg/dL)	2.58	< 0.14
NT-proBNP (pg/mL)	5466	< 125
PT-INR	0.96	
APTT (second)	29.4	26.9-38.1
D-dimer (μg/mL)	32	< 1.0
Antithrombin Ⅲ (%)	111	80-130
Protein C (%)	73	64-146
Protein S (%)	86	67-164
Venous lactic acid (mmol/L)	6.3	0.5-1.6

**Figure 1:**
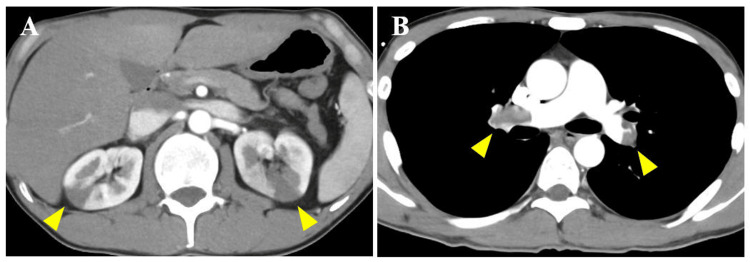
Contrast-enhanced computed tomography and pulmonary CT angiography (A) Abdominal contrast-enhanced computed tomography (CT) revealed bilateral renal infarctions (yellow arrowhead). (B) Pulmonary CT angiography revealed bilateral pulmonary embolisms (yellow arrowhead).

**Figure 2:**
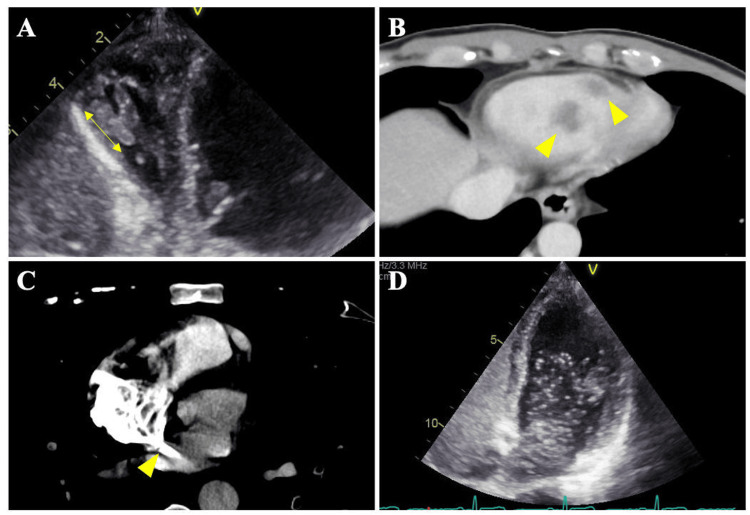
Cardiac imaging (A) Point-of-care ultrasound showed a mobile right ventricular thrombus (RVT) with a length of 15 mm. (B) Chest contrast-enhanced computed tomography (CT) revealed multiple RVT (yellow arrowhead). (C) Chest contrast-enhanced CT revealed contrast agent flow from the right atrium to the left atrium, suggesting a shunt through a patent foramen ovale (PFO) (yellow arrowhead). (D) Transthoracic echocardiography showed multiple bubbles in the left atrium and ventricle through the PFO.

We suspected acute mesenteric ischemia based on his symptoms and elevated lactic acid levels, as well as renal infarction due to an increased LDH. Thus, we utilized contrast-enhanced computed tomography (CT) in the late arterial and equilibrium phases to determine the cause. Contrast-enhanced CT of the abdomen revealed bilateral renal infarctions without dissection of the renal arteries (Figure 1A). Point-of-care ultrasound was performed to investigate the etiologies of renal infarctions and hypoxia, and it showed right ventricular dilation, right ventricular strain, tricuspid regurgitant pressure gradient above 50 mmHg, and a mobile thrombus at the apex of the right ventricle (Figure 2A). We suspected PE with paradoxical embolism and performed a pulmonary CT angiography and a lower extremity venous CT angiography, which showed thrombi in the bilateral main pulmonary arteries (Figure 1B), deep vein thrombosis at the left femoral vein, RVT (Figure 2B), and contrast agent flow from the right atrium to the left atrium, suggesting a shunt through a PFO (Figure 2C).

Based on the history of exertional dyspnea over the past few months, right ventricular enlargement, and an elevated tricuspid regurgitant pressure gradient (i.e., greater than 50 mmHg) [5], we made a diagnosis of acute-on-chronic PE complicated with renal infarctions through a PFO and RVT due to right ventricular dysfunction. After admission to the intensive care unit (ICU), he had hemodynamic stability and stable oxygen saturation with a high volume of supplemental oxygen. However, he had severe right ventricular dysfunction and a markedly elevated NT-proBNP level with significant oxygen demand and elevated heart rate and also had RVT. Based on his clinical status and the opinions of the cardio-vascular specialist, we initiated treatment of him with intravenous monteplase 800,000 IU and apixaban 10 mg orally twice daily. Prior to the initiation of thrombolytic therapy, a 4Fr sheath was inserted into both the right femoral artery and vein with extracorporeal membrane oxygenation (ECMO) on standby. This precaution was taken due to the risk of worsening PE resulting from the dislodgement of the thrombus in the RV during the therapy [6]. After starting treatment, his vital signs gradually improved without complications. He spent four days in the ICU, and he was orally administered apixaban 10 mg for one week, followed by a reduced dosage of 5 mg twice daily for two weeks. Follow-up transthoracic echocardiography (TTE) showed that dilatation of the RV had improved and the thrombus in the RV had disappeared and showed the absence of an atrial septal defect and left-to-right shunt. In addition, a micro-bubble study on TTE was positive during the Valsalva maneuver (Figure 2D).

TTE revealed no atrial septal aneurysm and Eustachian valve. Magnetic resonance imaging of the head did show any signals consistent with an ischemic stroke. Blood examinations showed that IgG of anticardiolipin antibody, IgG of anti-β2 glycoprotein I antibody, and lupus anticoagulant functional coagulation assay were negative. Two weeks after admission, a follow-up contrast-enhanced CT revealed that pulmonary artery thrombi were reduced. After a 21-day hospitalization, he was prescribed 15 mg of rivaroxaban once daily and was discharged without complications. He was treated with PFO disclosure six months after discharge.

## Discussion

The course of this patient suggested two important clinical issues: 1) PE patients with a PFO may initially present with a symptom due to an arterial thrombus rather than that due to a venous thrombus, and 2) PE may be accompanied by RVT, which can complicate the management of the condition.

Diagnosis of PE is difficult due to the inadequate sensitivity and specificity of symptoms and physical findings [7]. We should always consider this condition in differential diagnosis because PE is fatal when overlooked. Patients with PE typically present with symptoms associated with a venous thrombus (e.g., dyspnea, chest pain, or edema in the legs) at an emergency department [8]. However, if PE in patients with a PFO progresses sub-clinically, symptoms associated with arterial embolism through the PFO may be present initially, as in our case. The most important complication of arterial embolism is acute ischemic stroke, but, in rare cases, acute myocardial infarction, peripheral arterial embolism, and renal infarction may also occur [9]. Renal infarction is often caused by thromboembolism during atrial fibrillation or by renal artery dissection [10]. Although renal infarction caused by a paradoxical embolus is very rare, paradoxical embolism may cause multiple organ infarctions and bilateral renal infarction [11]. In such cases, we should investigate rare etiologies, especially etiologies such as PFO. Atrial septal aneurysm and the presence of the Eustachian valve and the Chiari network are risk factors for paradoxical embolism through a PFO [12,13]. It may be necessary to investigate which factors, such as patient characteristics or conditions, are associated with paradoxical embolism through a PFO.

In our case, we discovered PE with a PFO in the wake of the presence of bilateral renal infarctions. If patients with a diagnosis of PE do not have apparent arterial embolism, we may not consider searching for a PFO. However, in patients with PE, the presence of a PFO is known to be associated with thromboembolic complications and a high mortality rate [4]. Considering the relatively high prevalence of a PFO, it is concerning that a right-left shunt through a PFO could be present in all patients with PE, even those without arterial embolism. Especially in cases of acute-on-chronic PE, such as our case, right-left shunt through a PFO is likely to be caused by increased right ventricular and right atrial pressures.

Another teaching aspect about PE and PFO in our case is that the presence of a PFO was suspected based on findings of contrast-enhanced CT. Although a PFO is usually diagnosed by echocardiography with bubble study during the Valsalva maneuver, it has also been reported that contrast-enhanced CT could detect a PFO [14]. A pause of breath during a CT scan is thought to be the same situation as the Valsalva maneuver. In addition, in cases of PE, an increase in pulmonary artery pressure caused by a pulmonary embolus may lead to an increase in right atrial pressure and subsequently opening a PFO [15]. Thus, we should pay attention to the intracardiac structure, including a PFO when examining images of contrast-enhanced CT, especially in patients with PE.

RVT is a rare but important complication in patients with PE. This complication is often caused by severe PE with dilatation of the RV, as in our case, rather than mild PE [16]. The mechanism of the development of RVT is hypothesized to be PE-induced right heart failure and thrombus in the right ventricle derived from deep vein thrombosis [17]. This rare complication of PE has two important aspects. First, the thrombus must be distinguished from a tumor in the RV. Second, the administration of thrombolytics or anticoagulants may cause deterioration of PE by dislodging some of the thrombi in the RV and subsequently flying it to the pulmonary artery [16]. In patients with PE and coexisting RVT, reperfusion therapy is not associated with a reduced risk of PE-related mortality compared with no reperfusion therapy [18]. However, this evidence does not suggest that thrombolytic therapy for PE and coexisting RVT is safer than standard anticoagulant therapy [19]. Thus, in patients with concurrent PE and RVT, as in our case, it may be prudent to pre-cannulate (e.g., a 4 Fr sheath) for ECMO prior to initiating anticoagulation therapy. This approach considers the potential for RVT to worsen PE and lead to circulatory collapse. Although RVT is a rare complication of PE, this condition can be fatal if the thrombus in the right ventricle migrates to the pulmonary artery. Thus, patients with PE may require evaluation for RVT prior to thrombolytic or anticoagulant therapy.

## Conclusions

We describe a patient who presented with acute abdominal pain and was diagnosed with bilateral renal infarction. However, the condition was ultimately identified as paradoxical embolism through a PFO, which became apparent due to PE. The clinical presentation of PE can be diverse, and diagnosis can be challenging, especially in patients with arterial thrombosis associated with PFO. In this case, the patient also had RVT secondary to right ventricular dysfunction caused by PE. It is crucial to understand that the presence of RVT in PE patients can complicate anticoagulation therapy, potentially exacerbating PE due to the therapy. Thus, recognizing the possibility of concurrent PFO and RVT is important in the assessment of patients with PE, due to the complexity of the clinical manifestations and management.

## References

[1] Stein PD, Beemath A, Matta F (2007). Clinical characteristics of patients with acute pulmonary embolism: data from PIOPED II. Am J Med.

[2] Meissner I, Whisnant JP, Khandheria BK (1999). Prevalence of potential risk factors for stroke assessed by transesophageal echocardiography and carotid ultrasonography: the SPARC study. Mayo Clin Proc.

[3] Dao CN, Tobis JM (2011). PFO and paradoxical embolism producing events other than stroke. Catheter Cardiovasc Interv.

[4] Le Moigne E, Timsit S, Ben Salem D (2019). Patent foramen ovale and ischemic stroke in patients with pulmonary embolism: a prospective cohort study. Ann Intern Med.

[5] Opitz I, Patella M, Lauk O (2022). Acute on chronic thromboembolic pulmonary hypertension: case series and review of management. J Clin Med.

[6] Konstantinides SV, Meyer G, Becattini C (2020). 2019 ESC Guidelines for the diagnosis and management of acute pulmonary embolism developed in collaboration with the European Respiratory Society (ERS). Eur Heart J.

[7] Stein PD, Terrin ML, Hales CA, Palevsky HI, Saltzman HA, Thompson BT, Weg JG (1991). Clinical, laboratory, roentgenographic, and electrocardiographic findings in patients with acute pulmonary embolism and no pre-existing cardiac or pulmonary disease. Chest.

[8] Wicki J, Perneger TV, Junod AF, Bounameaux H, Perrier A (2001). Assessing clinical probability of pulmonary embolism in the emergency ward: a simple score. Arch Intern Med.

[9] Delalu P, Ferretti GR, Bricault I, Ayanian D, Coulomb M (2000). Paradoxical emboli: demonstration using helical computed tomography of the pulmonary artery associated with abdominal computed tomography. Eur Radiol.

[10] Antopolsky M, Simanovsky N, Stalnikowicz R, Salameh S, Hiller N (2012). Renal infarction in the ED: 10-year experience and review of the literature. Am J Emerg Med.

[11] Carey HB, Boltax R, Dickey KW, Finkelstein FO (1999). Bilateral renal infarction secondary to paradoxical embolism. Am J Kidney Dis.

[12] Cabanes L, Mas JL, Cohen A (1993). Atrial septal aneurysm and patent foramen ovale as risk factors for cryptogenic stroke in patients less than 55 years of age. A study using transesophageal echocardiography. Stroke.

[13] Schneider B, Hofmann T, Justen MH, Meinertz T (1995). Chiari's network: normal anatomic variant or risk factor for arterial embolic events?. J Am Coll Cardiol.

[14] Zhang M, Tan S, Patel V (2018). Patent foramen ovale in patients with pulmonary embolism: a prognostic factor on CT pulmonary angiography?. J Cardiovasc Comput Tomogr.

[15] Pinsky MR (2016). The right ventricle: interaction with the pulmonary circulation. Crit Care.

[16] Barrios D, Rosa-Salazar V, Jiménez D (2016). Right heart thrombi in pulmonary embolism. Eur Respir J.

[17] Finlayson GN (2008). Right heart thrombi: consider the cause. Can J Cardiol.

[18] Bikdeli B, Jiménez D, Muriel A, Barrios D, Ballaz A, Verhamme P, Monreal M (2020). Association between reperfusion therapy and outcomes in patients with acute pulmonary embolism and right heart thrombi. Eur Respir J.

[19] Zieliński D, Zygier M, Dyk W (2023). Acute pulmonary embolism with coexisting right heart thrombi in transit-surgical treatment of 20 consecutive patients. Eur J Cardiothorac Surg.

